# Kinematic gait differences in preschool children with autism spectrum disorder

**DOI:** 10.3389/fpsyg.2025.1652594

**Published:** 2026-04-29

**Authors:** Liria Akie Okai-Nóbrega, Thiago Ribeiro Teles Santos, Priscila Albuquerque Araújo, Clarissa Cardoso dos Santos Couto Paz, Bruna Avelar, Letícia Paes Silva, Alice Brochado Campolina, Ana Carolina Rodrigues Esteves de Rezende, Débora Marques de Miranda, Marco Romano Silva, Hani Camille Yehia, Adriano Vilela Barbosa, Ana Paula Pereira Lage, Sérgio Teixeira Fonseca

**Affiliations:** 1 Graduate Program in Rehabilitation Sciences, Physical Therapy School, Universidade Federal de Minas Gerais (UFMG), Belo Horizonte, Brazil; 2 Anamê Science and Technology in Children’s Health, Belo Horizonte, Brazil; 3 Faculty of Physical Education and Physical Therapy, Universidade Federal de Uberlândia, Uberlândia, Brazil; 4 Universidade de Brasília (UnB), Brasília, Brazil; 5 Graduate Program in Sports Sciences, Physical Education School, Universidade Federal de Minas Gerais (UFMG), Belo Horizonte, Brazil; 6 Graduate Program in Nuclear Medicine, Faculty of Medicine, Universidade Federal de Minas Gerais (UFMG), Belo Horizonte, Brazil; 7 Graduate Program in Electrical Engineering, Eletrical Engineering School, Universidade Federal de Minas Gerais, Belo Horizonte, Brazil

**Keywords:** autism spectrum disorder, movement analysis, gait, kinematics, children

## Abstract

**Background:**

Autism Spectrum Disorder (ASD) is primarily characterized by differences in communication and social interaction, but motor impairments are also commonly observed, often emerging in early childhood. Understanding these motor characteristics may contribute to earlier identification and intervention. This study aimed to compare walking kinematics between autistic and non-autistic preschool-aged children.

**Methods:**

This observational cross-sectional study included 20 children aged 3 to 4 years (10 autistic and 10 non-autistic). Autism diagnoses were confirmed using DSM-5 criteria and the Childhood Autism Rating Scale (CARS). Gait data were collected using a three-dimensional motion capture system with 41 passive markers as children walked independently over a 6-meter walkway at a self-selected speed. Spatiotemporal and kinematic parameters were analyzed using independent t-tests, Mann–Whitney U tests, and one-dimensional statistical parametric mapping (SPM). Statistical significance was set at *p* < 0.05.

**Results:**

No significant differences were found between groups in spatiotemporal gait parameters. However, autistic children exhibited significantly greater hip abduction compared to non-autistic children at the beginning and end of the gait cycle.

**Conclusion:**

Differences in frontal plane hip kinematics may reflect a gait motor “signature” associated with autism in early childhood. These findings support the potential use of motion analysis as a quantitative tool to aid early identification and intervention strategies for autistic children.

## 1 Introduction

Autism Spectrum Disorder (ASD) is a neurodevelopmental condition characterized by differences in social communication and interaction, alongside restricted and repetitive patterns of behavior, interests, or activities (American Psychiatric Association, 2013). While ASD is primarily diagnosed based on social and behavioral characteristics, research increasingly highlights the presence of motor impairments in autistic individuals, including in early childhood (Bhat, 2020; Lum et al., 2021; Ayoub et al., 2025). These motor differences, including higher gait variability even when average measures appear typical, may serve as early markers of autism and reflect altered motor control strategies. Beyond gross motor skills, oromotor impairments affect speech and feeding (Maffei et al., 2023), illustrating how motor function influences broader developmental domains such as language and social communication. These motor differences, though not included in the core diagnostic criteria, may offer critical insight into early developmental trajectories and potentially serve as early markers of autism.

Motor impairments are highly prevalent in the autistic population, with estimates ranging from 67.4 to 86.9% (Reynolds et al., 2022; Bhat, 2020), and are comparable in impact to difficulties in language and adaptive functioning. Difficulties with balance, coordination, posture, gait, strength, and agility have been documented and are also associated with differences in social communication (Casartelli et al., 2016). A growing body of research supports the connection between motor and social development in autism, with evidence suggesting that interventions targeting motor skills may yield benefits in speech and social engagement (Odeh et al., 2020; Gladfelter et al., 2020).

Gait analysis, in particular, has emerged as a promising approach for identifying motor differences in autistic children. Prior studies have explored spatiotemporal features (e.g., step width, stride length, walking speed) and kinematic variables (e.g., joint range of motion) in autistic versus non-autistic children (Calhoun et al., 2011; Kindregan et al., 2015; Jequier Gygax et al., 2021). However, findings remain inconsistent. Some studies report wider steps, slower speeds, and altered timing in autistic children (Eggleston et al., 2020; Lum et al., 2021), while others find no significant group differences (Dufek et al., 2017; Kindregan et al., 2015). These discrepancies may stem from the wide heterogeneity within the autism spectrum, small and varied samples, methodological differences, and the frequent focus on school-aged rather than preschool-aged populations (Lum et al., 2021).

To address these gaps, the current study focuses specifically on gait kinematics in autistic and typically developing (TD) children aged 3 to 4 years, a developmental window where early identification of autism is particularly impactful for access to intervention. By limiting age variation and employing a high-resolution three-dimensional motion analysis system, this study aims to clarify whether kinematic or spatiotemporal differences exist in early childhood gait patterns. We hypothesized that autistic children would show distinct gait features, particularly in joint kinematics, compared to their TD peers, potentially offering useful markers for earlier autism detection.

## 2 Methods

### 2.1 Participants

This observational cross-sectional study included autistic and typically developing (TD) children aged 3 to 4 years. A total of 25 children (23% female; mean age = 47.78 ± 8.69 months; height = 1.05 ± 0.07 m; body mass = 17.97 ± 4.89 kg) were initially recruited through convenience sampling via community advertisements. Five participants were excluded due to insufficient gait data (i.e., fewer than 14 valid steps), resulting in a final sample of 20 children: 10 autistic boys and 10 age-matched TD children (8 boys, 2 girls).

Inclusion criteria comprised: (1) age between 3 and 4 years, (2) ability to follow instructions, (3) ability to walk independently, and (4) verbal or non-verbal communication abilities. Exclusion criteria included: (1) chronic medical conditions other than ASD, (2) orthopedic or neurological impairments affecting gait, (3) aggressive behaviors, or (4) refusal to participate in any procedures.

All autistic participants had a clinical diagnosis of ASD confirmed by a specialist in child neuropsychiatry with over 10 years of experience, based on DSM-5 criteria. TD children were recruited via pediatricians and kindergartens and had no history of developmental, neurological, or communication disorders.

This study was approved by the University Research Ethics Committee (approval number: 2.083.328), and written informed consent was obtained from all parents or legal guardians before participation.

### 2.2 Instrumentation and procedures

**Figure 1:**
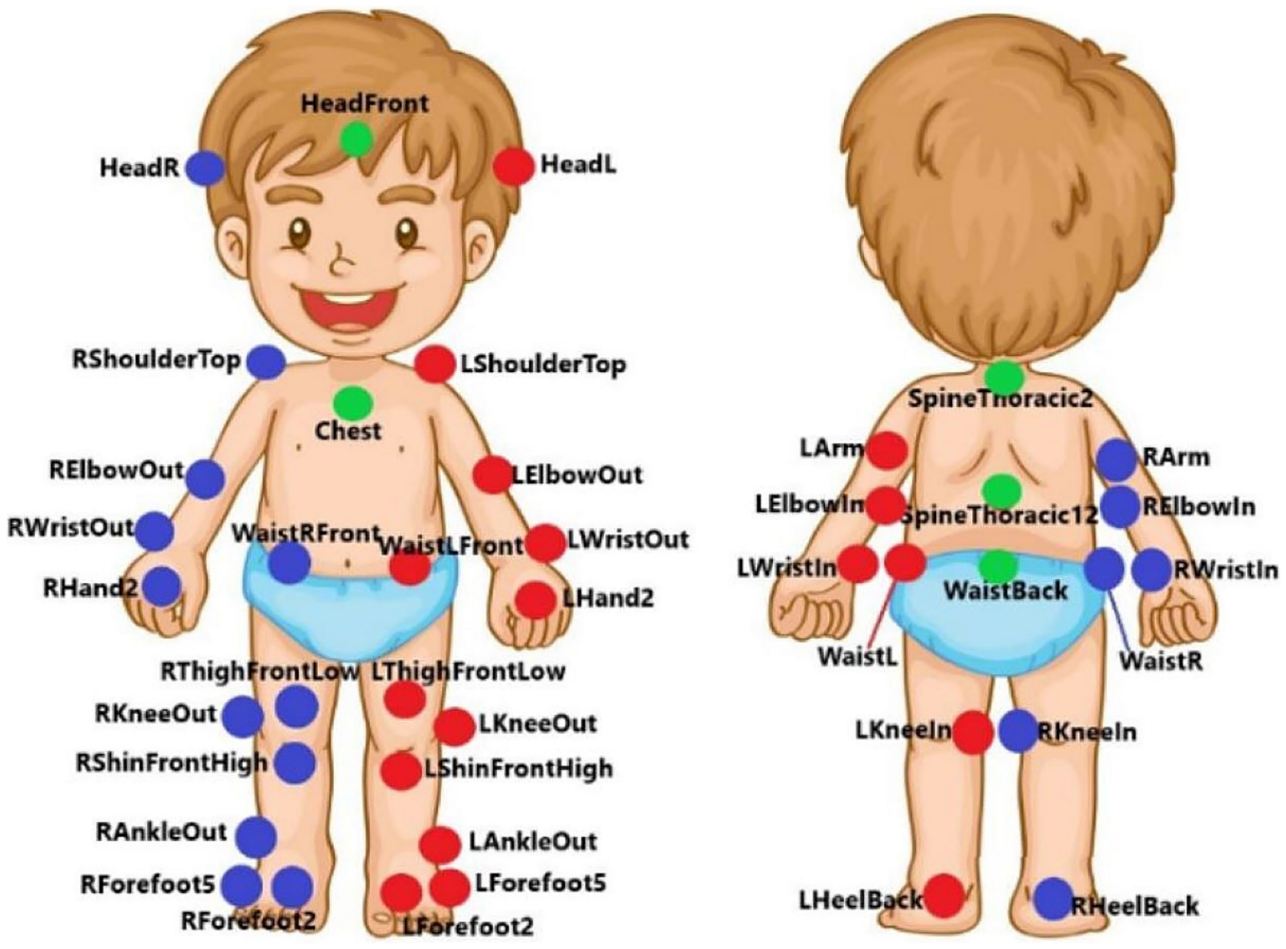
Setup of the marker set. Red dots represent left-side markers, blue, right-side markers, and green, central markers. Forty-one passive markers were placed on: glabella (HeadFront); jugular notch (Chest); 2 cm over ear lobe directing to acoustic meatus (HeadR and HeadL); T2 (SpineTroracic2), T12 (SpineThoracic12), acromioclavicular joint (RShoulderTop and LShoulderTop); lateral and medial epicondyle of the humerus (RElbowOut, LElbowOut, RElbowIn, LElbowIn); triceps musculotendinous junction (RArm and Larm); radial and ulnar styloid process (RWristIn, LWristIn, RWristOut and LWristOut); Sacrum (WaistBack); 2nd metacarpal head (RHand2 and LHand2); anterior superior iliac spine (WaistRFront and WaistLFront); iliac crest (WaistR and WaistL); lateral and medial condyle of femur (RKneeOut, LKneeOut, RKneeIn and LKneeIn); 2 cm over patella base (RThighFrontLow and LThighFrontLow); tibial tuberosity (RShinFrontHigh and LShinFrontHigh); lateral malleolus (RAnkleOut and LAnkleOut); Calcaneus (RHeelBack and LHeelBack); 2nd metatarsal head (RForefoot2 and LForefoot2); and 5th metatarsal head (RForefoot5 and LForefoot5) (Bell et al., 1989; Tranberg, 2010; Wu et al., 2002, 2005).

Three-dimensional kinematic data were captured using ten infrared cameras (Qualisys Oqus VII, Qualisys Medical AB, Gothenburg, Sweden). A total of 41 passive reflective markers were placed bilaterally at specific anatomical landmarks following a full-body pediatric marker set (see Figure 1).

ASD diagnoses were confirmed through clinical interviews and Childhood Autism Rating Scale (CARS), a 15-item behavioral scale with scores >30 indicating autism (Moon et al., 2019; Pereira et al., 2008) and characterized by Snijders-Oomen Non-verbal Intelligence Test (SON-R), assesses visuomotor reasoning and abstract thinking for children aged 2.5–7 years (Laros et al., 2013) and Brazil Economic Classification Criteria (BECC), evaluates socioeconomic status based on household assets, education level, and access to services [Associação Brasileira de Empresas de Pesquisa (ABEP), 2022].

Anthropometric measurements (height and weight) and developmental history were collected for all children. For autistic participants, additional information on age and time of diagnosis was recorded. Lower limb dominance was determined using a ball-kicking task (Polk et al., 2017).

**Figure 2:**
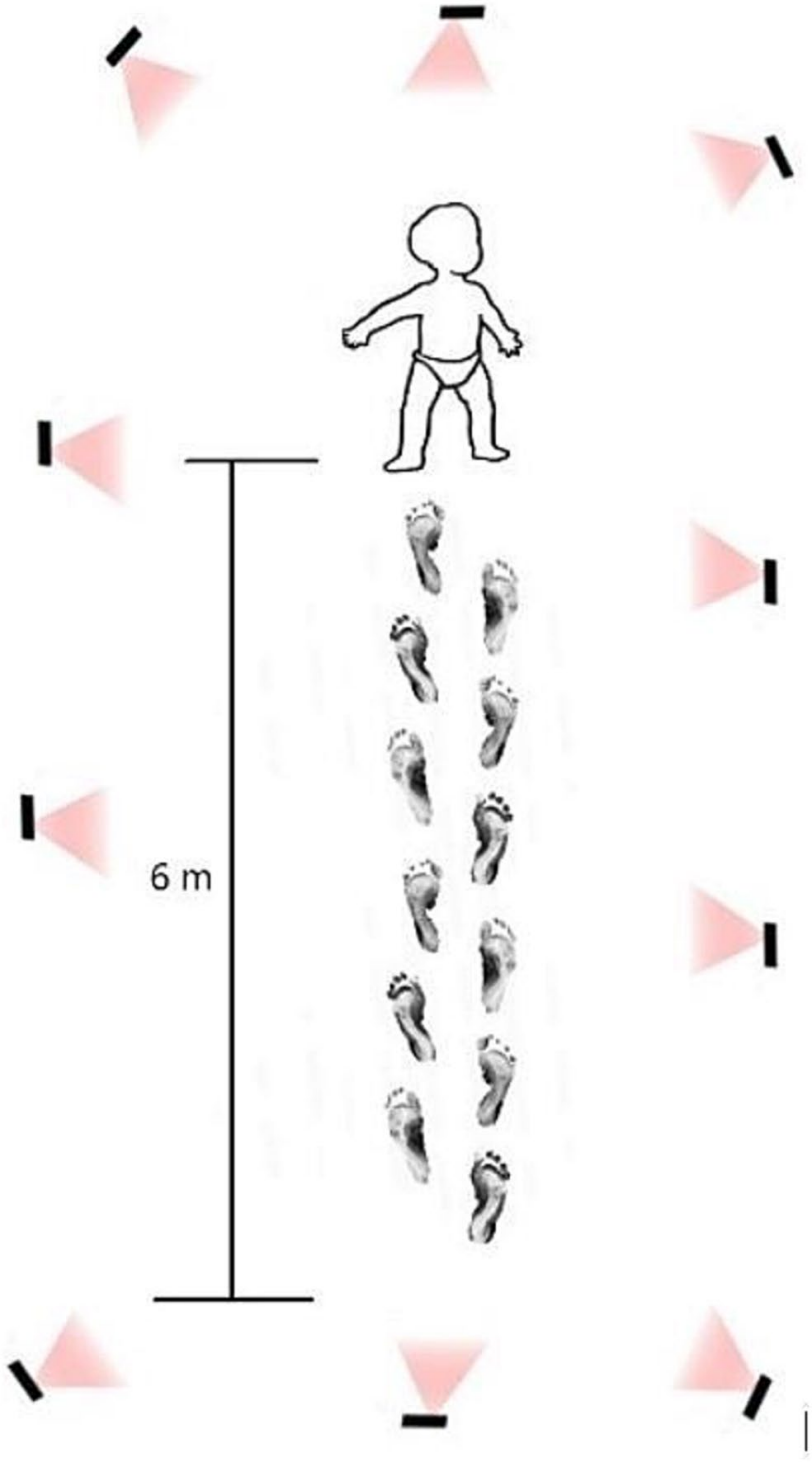
Laboratory settings: children were instructed to walk on a 6 m area with ten infrared cameras for three minutes.

Before walking trials, each child stood still for 5 s in a quiet stance to calibrate the neutral position of the kinematic model. Participants were then asked to walk at a self-selected pace along a 6-meter walkway. At least three walking trials, each lasting approximately three minutes, were conducted per child (see Figure 2).

### 2.3 Data reduction and analysis

Marker trajectories were processed using Qualisys Track Manager software (Qualisys Medical AB, Sweden) and further analyzed in Visual3D (C-Motion, Inc., Germantown, MD, United States). Marker displacement data were filtered with a fourth-order low-pass Butterworth filter (cut-off frequency: 6 Hz). Trials were retained only if the child walked continuously without unrelated voluntary movements. Fourteen consecutive steps from the middle portion of each trial were analyzed.

Initial contact and toe-off events were detected using the algorithm described by Borghese et al. (1996), later adapted for children by Ivanenko et al. (2004) and applied in toddler gait analysis (Santos et al., 2022). Spatiotemporal parameters extracted included: cycle time, stance time, double support time, swing time, cadence, walking speed, step length, stride length, and step width. Kinematic parameters (pelvis, hip, knee, and ankle angles) were analyzed in the sagittal, frontal, and transverse planes, based on a medial-lateral, anterior–posterior, and vertical Cardan rotation sequence. Angle time series were time-normalized to 101 data points per gait cycle.

Descriptive statistics were computed for group characteristics and spatiotemporal parameters. Data normality was assessed using the Shapiro–Wilk test. For normally distributed variables (e.g., height, limb length, spatiotemporal parameters), independent samples *t*-tests were performed. Effect sizes were calculated using Cohen’s *d* and interpreted as small (0.20), medium (0.50), or large (0.80) (Cohen, 1988). For non-normally distributed variables (e.g., age, body mass, CARS, SON-R, BECC), Mann–Whitney *U* tests were used. Chi-square tests with continuity correction were applied for categorical variables.

Kinematic curves were compared using one-dimensional statistical parametric mapping (SPM). Independent SPM *t*-tests were used to compare joint angle trajectories (pelvis, hip, knee, ankle) between groups across the gait cycle. The D’Agostino–Pearson *K^2^* test was used to assess normality within the SPM analysis. The resulting SPM[*t*] statistic was compared to the critical threshold (*α* = 0.05). When significant differences were found, the timing within the gait cycle and corresponding *p*-values were reported. Effect sizes were calculated using Hedges’ *g* and interpreted as small (0.20–0.49), medium (0.50–0.79), or large (≥0.80) (Lakens, 2013).

## 3 Results

Table 1 presents the characteristics of the autism spectrum disorder (ASD) and Typically Developing (TD) groups. No significant differences were found between groups in age, height, lower limb length, or body mass. However, the groups differed significantly in scores related to autism characteristics (CARS), non-verbal intelligence (SON-R), and socioeconomic classification (BECC).

**Table 1:** Characteristics of ASD and TD group.

Variables	ASD group, *n* = 10	TD group, *n* = 10	*p*-value
Age, median (IQR)	54.5 (10.8) months	52.5 (11.0) months	0.850
Height, mean (SD)	1.08 (0.06) m	1.05 (0.06) m	0.289
Lower limb length, mean (SD)	0.52 (0.05) m	0.49 (0.04)	0.135
Body mass, median (IQR)	18.40 (4.93) kg	16.75 (1.68) kg	0.130
CARS			
Score, median (IQR)	35.65 (4.15)	17.80 (1.30)	< 0.001*
SON-R			
SON-IQ			
Score, median (IQR)	100.00 (22.00)	120.50 (10.00)	0.002*
SON-ES			
Score, mean (SD)	94.00 (20.29)	115.20 (9.67)	0.009*
SON-RE			
Score, mean (SD)	95.44 (16.70)	118.40 (8.76)	0.001*
BECC			
Score, mean (SD)	28.60 (8.80)	47.40 (10.19)	< 0.001*

ASD = Autism Spectrum Disorder, TD = Typical developing, *n* = number of participants, IQR = Interquartile range, SD = Standard deviation, CARS = Childhood Autism Rating Scale, SON-R = Snijders-Oomen Non-Verbal Intelligence Test, IQ = Intelligence quotient, ES = Execution scale, RS = Reasoning scale, BECC = Brazil Economic Classification Criteria developed by the Associação Brasileira de Empresas de Pesquisa. *statistical difference.

Spatiotemporal walking parameters did not differ significantly between the ASD and TD groups (Table 2). Effect sizes ranged from negligible to small. All comparison showed statistical power lower than 0.379.

**Table 2:** Spatiotemporal walking parameters.

Variables	ASD group, *n* = 10	TD, *n* = 10	*p*-value	Cohen’s d	Power
Cycle time (s)					
DOM	0.837 (0.119)	0.840 (0.077)	0.934	0.030	0.050
NDOM	0.277 (0.034)	0.284 (0.023)	0.565	0.241	0.080
Swing time (s)					
DOM	0.282 (0.030)	0.293 (0.022)	0.385	0.418	0.144
NDOM	0.277 (0.034)	0.284 (0.023)	0.565	0.241	0.080
Stance time (s)					
DOM	0.552 (0.091)	0.550 (0.057)	0.954	0.026	0.050
NDOM	0.552 (0.105)	0.559 (0.054)	0.860	0.084	0.054
Cadence (steps/min)					
DOM	148.686 (24.455)	143.091 (12.271)	0.526	0.289	0.094
NDOM	150.010 (26.827)	145.607 (13.204)	0.647	0.208	0.073
Step length (m)					
DOM	0.400 (0.069)	0.407 (0.051)	0.797	0.115	0.057
NDOM	0.379 (0.057)	0.401 (0.056)	0.398	0.389	0.131
Stride length (m)					
DOM	0.778 (0.120)	0.809 (0.092)	0.527	0.290	0.094
NDOM	0.777 (0.119)	0.807 (0.096)	0.536	0.277	0.090
Stride width (m)	0.135 (0.029)	0.117 (0.015)	0.094	0.780	0.379
Speed (m/s)	0.961 (0.216)	0.966 (0.144)	0.959	0.027	0.050

Data are presented as Mean (Standard Deviation). *n* = Number of participants, DOM = Dominant lower limb, NDOM = Non-dominant lower limb.

**Figure 3:**
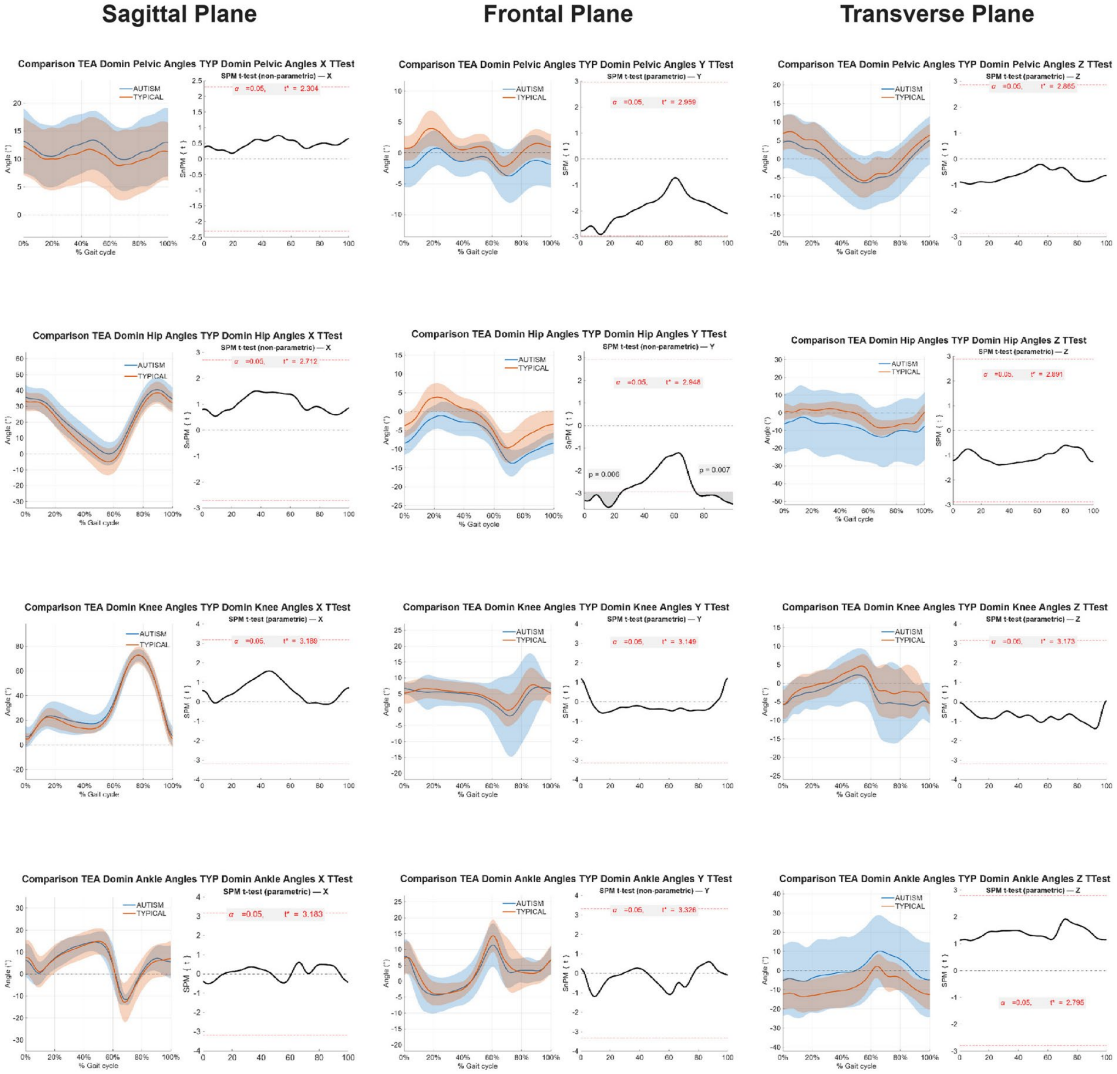
The 1-dimensional statistical parametric mapping (SPM) independent t-test parameters comparing Autism Spectrum Disorder (ASD) and typical developing (TD) children during the gait cycle (normalized with 101 points). The dashed line represents the average curve of joint kinematics of the ASD group, and the continuous line represents the average joint kinematics of the TD group. The light and dark shaded areas represent standard deviation curves of TD and ASD, respectively. In the sagittal plane plots, positive angles indicate pelvis retroversion, hip and knee flexion, and ankle dorsiflexion. In the frontal plane’s plots, positive angles indicate pelvis uptilt, hip and knee adduction, and ankle inversion. In the transverse plane plots, positive angles indicate pelvis, hip, and knee medial rotation, and ankle adduction. Angles indicate pelvis, hip, and knee medial rotation, and ankle adduction.

Significant differences were observed in the frontal plane hip kinematics between groups. Children with ASD exhibited greater hip abduction than TD children during two distinct portions of the gait cycle: the loading response (0–35%) and terminal swing (73–100%) phases (Figure 3). No significant group differences were found in other joints or movement planes.

## 4 Discussion

This study compared walking kinematics between preschool-aged children with autism spectrum disorder (ASD) and their typically developing (TD) peers. The key finding was that children with ASD exhibited significantly greater hip abduction in the frontal plane during two specific periods of the gait cycle: 0–35% (early stance) and 73–100% (terminal swing). These findings differ from previous reports in school-aged children, which identified group differences at toe-off (Nobile et al., 2011), suggesting that gait alterations in ASD may be age-dependent.

The increased hip abduction observed in the ASD group may reflect compensatory strategies for lateral balance control, as suggested by earlier work linking greater hip abduction to wider step width (Lum et al., 2021) and to stabilization demands during single-limb support (Vistamehr and Neptune, 2021). This strategy may also be related to hypotonia, commonly reported in individuals with ASD, which can delay motor development, impair postural control, and reduce movement precision (Galli et al., 2011; Ha and Sung, 2022; Horlings et al., 2009). Interventions addressing hypotonia and motor coordination may therefore improve gait stability and functional mobility in young children on the autism spectrum.

Despite the hip kinematic differences, no group differences were observed in spatiotemporal parameters or other joint kinematics. This aligns with prior findings suggesting that gross motor patterns in ASD may not consistently diverge from typical development (Calhoun et al., 2011). Nevertheless, motor impairments are reported in approximately 79% of autistic children and are associated with reduced quality of life and social participation (Lai et al., 2014). Given the importance of walking as a fundamental motor activity linked to independence and social integration, early identification of gait differences may have clinical relevance.

This study has limitations. The sample size was small, limiting generalizability. However, this is one of the first studies to explore these kinematic gait differences, and it may serve as a foundation for future research aiming to replicate and expand these findings in larger samples. Additionally, the limited sample size and small effect size affected the statistical power for the spatiotemporal parameters. Multiple parameters showed equal or nearly equal means and standard deviations between groups, resulting in a small effect size. The restricted sample size limited the argument of the absence of a difference between groups. On the other hand, the required sample size for the identified effect sizes for some parameters would be impractical to recruit and casts doubt if the difference really exists in the population. Furthermore, ASD subtypes were not differentiated, and standardized motor assessments were not included, restricting the ability to correlate observed gait differences with broader motor profiles. Additionally, the focus on preschool-aged children may explain discrepancies with studies involving older participants (Biffi et al., 2018; Eggleston et al., 2018). Our sample included only boys with ASD, and although the TD group was not fully sex-matched, no significant demographic or anthropometric differences were found (Table 1); nonetheless, the limited female representation restricts generalizability, underscoring the need for future studies with larger and more balanced cohorts. A major strength of this study, however, is the use of one-dimensional statistical parametric mapping (SPM), which enabled the identification of time-specific gait differences across the full gait cycle, rather than relying solely on discrete outcome measures (Biffi et al., 2018; Calhoun et al., 2011).

These findings underscore the potential of motion analysis for early identification of ASD-related motor differences. Subtle deviations such as increased hip abduction may serve as early motor markers, complementing behavioral assessments in diagnostic and intervention frameworks. Early detection and targeted motor interventions may enhance developmental trajectories and overall quality of life for autistic children. Future studies with larger samples and longitudinal designs are needed to validate and extend these findings.

Children on the autism spectrum exhibited greater hip abduction compared to their typically developing peers during walking. These motor differences, observable as early as the preschool years, suggest that gait analysis may complement behavioral assessments in early ASD identification. Beyond socio-communicative challenges, altered motor patterns highlight the potential of movement-based markers for early recognition and intervention in autism spectrum disorder.

## Data Availability

The raw data supporting the conclusions of this article will be made available by the authors, without undue reservation, upon reasonable request to the corresponding author.
